# Cardiometabolic index as a predictor of major adverse cardiovascular events in atrial fibrillation: insights from a community-based cohort

**DOI:** 10.3389/fendo.2025.1682622

**Published:** 2025-10-03

**Authors:** Jingjing Sha, Jiayue Cheng, Xunhan Qiu, Mangmang Pan, Caihong Liu, Long Shen, Zhichun Gu, Hao Huang, Siliang Zeng

**Affiliations:** ^1^ Jinyang Community Health Service Center, Shanghai, China; ^2^ Renji Hospital, Shanghai Jiao Tong University School of Medicine, Shanghai, China; ^3^ Department of Rehabilitation Therapy, Shanghai Normal University Tianhua College, Shanghai, China

**Keywords:** atrial fibrillation, cardiometabolic index, machine learning, major adverse cardiovascular events, risk prediction

## Abstract

**Background:**

The cardiometabolic index, a composite indicator integrating central obesity and lipid abnormalities, has demonstrated predictive value in several cardiovascular diseases. However, its role in predicting major adverse cardiovascular events among patients with atrial fibrillation remains underexplored.

**Methods:**

In this single-center retrospective cohort study, 192 atrial fibrillation (AF) patients under management at the Jinyang Community Health Service Center in Pudong, Shanghai, from January 2022 to January 2024 were enrolled. Patients were stratified into tertiles based on baseline cardiometabolic index (CMI). The primary endpoint was major adverse cardiovascular events (MACE), comprising cardiovascular death, nonfatal myocardial infarction, nonfatal stroke, hospitalization for worsening heart failure, and coronary revascularization due to unstable angina or ischemic events. Multivariable Cox proportional hazards models were used to assess the independent association between CMI and MACE. Kaplan–Meier curves and Log-rank tests were applied to compare event incidence across groups. Restricted cubic spline analysis examined potential nonlinearity. An extreme gradient boosting model was developed to evaluate predictive performance, with SHapley Additive exPlanations used to assess variable importance. Subgroup analyses were conducted to evaluate the consistency of CMI’s predictive value across different clinical populations. The median follow-up duration was 664 days (interquartile range: 384–900 days), estimated using the reverse Kaplan–Meier method.

**Results:**

MACE incidence increased significantly with rising CMI levels. Compared to the low CMI group, the high CMI group had a significantly higher risk of MACE (HR = 5.56, 95% CI: 1.48 – 20.90, P = 0.011). Kaplan–Meier analysis showed significant differences in cumulative incidence among the three groups (Log-rank P < 0.001). restricted cubic spline (RCS) modeling revealed a nonlinear positive association, with a sharp increase in MACE risk above a CMI threshold of approximately 0.85 (P for nonlinearity < 0.001). The Extreme Gradient Boosting (XGBoost) model achieved a C-index of 0.737 in the test set, with SHapley Additive exPlanations (SHAP) analysis ranking CMI as the fourth most influential predictor, following age, left atrial diameter, and left ventricular ejection fraction. Subgroup analyses suggested that the predictive value of CMI was particularly evident in patients without chronic kidney disease and those without prior catheter ablation.

**Conclusion:**

Elevated CMI is independently associated with increased MACE risk in patients with atrial fibrillation and demonstrates a nonlinear dose–response relationship. As a simple, accessible metabolic indicator, CMI shows promise for improving cardiovascular risk identification and guiding personalized management—especially in high-risk AF patients without overt metabolic dysfunction.

## Introduction

Atrial fibrillation (AF) is one of the most prevalent persistent cardiac arrhythmias worldwide, with its incidence rising rapidly among the aging population and posing a significant threat to cardiovascular health (1–3). AF not only elevates the risk of stroke, heart failure, and all-cause mortality, but also imposes a considerable burden on healthcare systems, making it a major global public health concern (4). Community health service centers play a pivotal role in the long-term management of patients with AF and serve as key channels for referral and escalation of care. In this context, the timely identification of high-risk individuals and the implementation of personalized interventions have become essential responsibilities in general practice to reduce AF-related complications.

In current clinical practice, the CHA_2_DS_2_-VASc score is commonly used to assess thromboembolic and major adverse cardiovascular event (MACE) risks in AF patients (5, 6). However, this score is primarily based on clinical variables such as age, hypertension, and diabetes, and does not adequately capture metabolic disturbances or changes in body composition, limiting its predictive performance in certain populations. Emerging evidence suggests that metabolic abnormalities—particularly central obesity and dyslipidemia—play a crucial role in the development and progression of AF (7–10). This underscores the need for novel metabolic markers to complement conventional risk models.

The cardiometabolic index (CMI)—a composite metric that integrates waist-to-height ratio and the triglyceride-to-high-density lipoprotein cholesterol ratio—reflects both central obesity and dyslipidemia. CMI has demonstrated robust predictive value in cardiovascular conditions such as coronary artery disease and heart failure (11–14). Nonetheless, systematic investigations into its association with MACE among patients with AF remain scarce, and its independent prognostic value and mechanistic underpinnings have yet to be clarified.

Accordingly, this study utilized a real-world, community-based cohort to systematically examine the association between CMI and the incidence of MACE in patients with AF. Specifically, we aimed to determine whether CMI independently predicts MACE and to explore potential non-linear associations. We further assessed the performance of CMI across different clinical subgroups and its incremental value when incorporated into traditional risk models. These findings are expected to provide a more comprehensive approach to identifying metabolic risk in AF patients and to support the advancement of individualized risk management in clinical practice.

## Methods

### Study design and population

This single-center, retrospective cohort study included patients with atrial fibrillation who received chronic disease management follow-up at the Jinyang Community Health Service Center in Shanghai from January 2022 to January 2024. The majority of participants were referred from the Department of Cardiology at Renji Hospital, Shanghai Jiao Tong University School of Medicine, and enrolled in the community-based high-risk population management platform of the regional medical consortium. These patients underwent regular, standardized clinical evaluations and laboratory tests.

Inclusion criteria were: (1) age ≥18 years; and (2) a confirmed diagnosis of AF according to the European Society of Cardiology (European Society of Cardiology (ESC)) guidelines (15), with enrollment in follow-up management. Exclusion criteria included: (1) failure to meet the ESC diagnostic criteria for AF; (2) interruption of follow-up or lack of at least one documented follow-up visit; (3) concomitant moderate to severe valvular heart disease, prior valve replacement surgery, or history of congenital heart disease surgery; (4) presence of active malignancy; and (5) missing key clinical or biochemical data, such as fasting blood glucose or triglyceride levels.AF type was classified as paroxysmal or persistent according to the 2024 ESC guideline definitions; the classification recorded at baseline was used for analysis.

**Figure 1:**
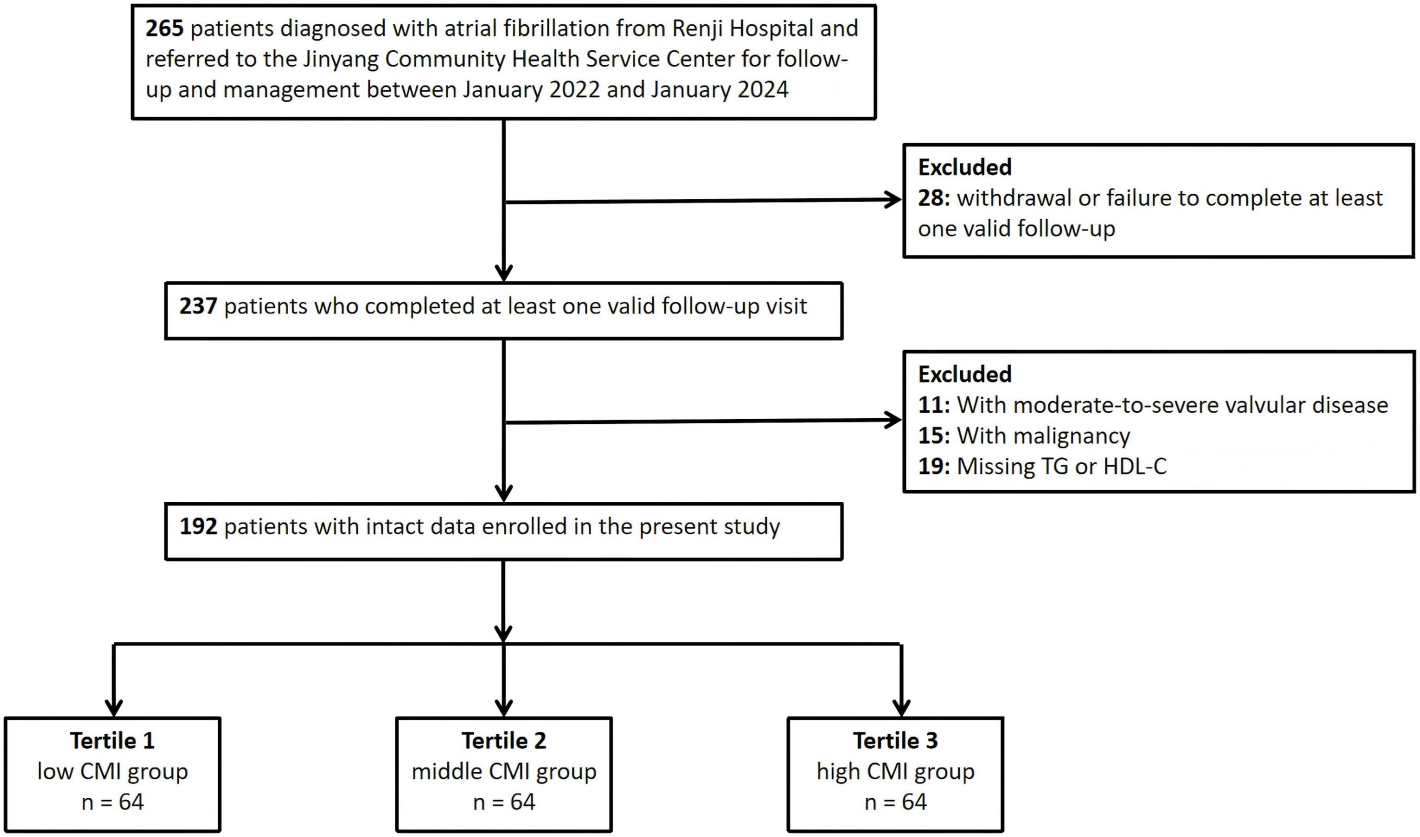
Flowchart of study population enrollment.

A total of 192 patients met the eligibility criteria and were included in the final analysis. The study flowchart is presented in **Figure 1**. The study protocol was approved by the Ethics Committee of Renji Hospital, Shanghai Jiao Tong University School of Medicine (Approval No.: KY2022-105-B) and adhered strictly to the Declaration of Helsinki and relevant ethical standards. As a retrospective study utilizing previously collected medical records, written informed consent was not obtained. However, the ethics committee granted a waiver of consent, considering the potential scientific and clinical value of the study to outweigh the minimal risk to individual privacy. All data were fully anonymized to protect patient confidentiality and ensure data security.

### Follow-up and outcome ascertainment

Follow-up and outcome data were obtained through scheduled clinic visits, structured telephone interviews, and linkage to the electronic health record system. The prespecified censoring date was January 31, 2024. The primary endpoint (MACE) comprised cardiovascular death, nonfatal myocardial infarction, nonfatal stroke, hospitalization for worsening heart failure, and coronary revascularization for unstable angina or ischemic events; all events were adjudicated by two cardiologists with a third reviewer resolving discrepancies. Median follow-up was 664 days (IQR 384–900), estimated by the reverse Kaplan–Meier method.

### Basic data collection

Basic clinical data for this study were sourced from the electronic health record system of the Jinyang Community Health Service Center. Demographic variables included age, sex, height, weight, body mass index (BMI), waist circumference, and hip circumference. Medical history variables included the presence of hypertension, diabetes mellitus, coronary artery disease (CAD), chronic kidney disease (CKD), heart failure, and stroke. Treatment-related information encompassed anticoagulation status and whether the patient had undergone radiofrequency ablation. Laboratory parameters included fasting plasma glucose, glycated hemoglobin, lipid profile components—triglycerides, high-density lipoprotein cholesterol (HDL-C), low-density lipoprotein cholesterol (LDL-C), and total cholesterol—as well as liver function indicators (Alanine Aminotransferase, Aspartate Aminotransferase, total bilirubin), renal function (serum creatinine), and complete blood count variables including hemoglobin, red blood cell count, hematocrit, and platelet count. Echocardiographic assessments included the left atrial anteroposterior diameter (LAID) and left ventricular ejection fraction (LVEF). Additionally, the following validated clinical scores and classifications were recorded: atrial fibrillation type, European Heart Rhythm Association (EHRA) symptom score, CHA_2_DS_2_-VASc score, and HAS-BLED score. The primary study endpoint was the occurrence of MACE, defined as a composite of cardiovascular death, nonfatal myocardial infarction, nonfatal stroke, hospitalization for worsening heart failure, or coronary revascularization prompted by unstable angina or other ischemic events. Follow-up duration was calculated in days from the date of enrollment to the first occurrence of a MACE or the end of the observation period. All data were independently extracted by two trained cardiologists. In cases of discrepancy, a third investigator was involved to review and adjudicate the data, ensuring accuracy and consistency.

### Cardiometabolic index formula

The Cardiometabolic index was calculated using the following formula:

CMI=WHtR * (triglycerides (TG) [mmol/L]/HDL-C [mmol/L]), WHtR = Waist Circumference (cm)/height (cm).

### Statistical analyses

All statistical analyses were conducted using R (v4.4.2) and Python (v3.11.8) within a Jupyter Notebook environment, leveraging packages including tidyverse, survival, rms, xgboost, sklearn, and SHAP. All tests were two-tailed, with P < 0.05 considered statistically significant and P < 0.01 highly significant. Preprocessing included variable selection, handling of missing values, and normality testing. Variables with >10% missing data were excluded. The Shapiro–Wilk test was used to assess normality. Normally distributed variables were reported as mean ± SD and compared via one-way ANOVA; non-normal variables as median (IQR) using the Kruskal–Wallis H test. Categorical variables were summarized as counts (%) and compared using the chi-square or Fisher’s exact test, as appropriate.

To assess the association between CMI and MACE, patients were grouped into CMI tertiles, with the lowest tertile as reference. Cox proportional hazards models were used. Model 1: unadjusted; Model 2: adjusted for age and sex; Model 3: further adjusted for comorbidities (hypertension, CAD, CKD, heart failure, prior stroke). Results were reported as HRs with 95% CIs. Trend analysis treated CMI tertiles as a continuous variable. Model discrimination was evaluated using Harrell’s C-index. Kaplan–Meier curves with log-rank tests were used for cumulative incidence comparison. Internal validation via 5,000 bootstrap iterations provided bias-corrected C-index estimates.

Restricted cubic splines (RCS) with three knots modeled potential non-linear associations between CMI and MACE, with Wald test assessing non-linearity. A machine learning model using XGBoost was developed with clinical and lab features as predictors and MACE as the outcome. Data were split 8:2 for training/testing. 10-fold cross-validation optimized hyperparameters. Discrimination was evaluated via C-index. SHAP values quantified each feature’s contribution. Subgroup analyses were conducted via multivariable Cox models across strata defined by age (≥65 vs. <65), sex, hypertension, CAD, CKD, heart failure, anticoagulation, and catheter ablation to assess consistency of CMI’s prognostic value.

## Results

### Baseline characteristics

A total of 192 patients with AF were included in the analysis. Based on baseline CMI levels, patients were divided into tertiles, with the lowest tertile serving as the reference group in Cox proportional hazards regression models. Baseline characteristics across the three CMI groups are summarized in **Table 1**. There were no statistically significant differences in age, height, weight, BMI, fasting plasma glucose, or glycated hemoglobin among the groups (all P > 0.05). However, waist circumference (P = 0.013) and hip circumference (P = 0.022) increased significantly with higher CMI levels, indicating a progressively stronger pattern of central obesity. Significant differences were also observed in metabolic parameters. Triglyceride levels were notably higher in the high CMI group compared to the low and middle groups (1.02 [0.85–1.20] mmol/L, 1.75 [1.57–1.88] mmol/L, and 2.31 [2.11–2.96] mmol/L, respectively; P < 0.01). In contrast, HDL-C levels declined markedly across tertiles (1.20 [1.16–1.26] mmol/L, 1.01 [0.96–1.06] mmol/L, and 0.82 [0.78–0.86] mmol/L, respectively; P < 0.01). Both total cholesterol and LDL-C also differed significantly among groups (P < 0.01 and P = 0.014, respectively). No significant intergroup differences were observed for hemoglobin levels or liver and renal function markers (all P > 0.05). During follow-up, the incidence of MACE varied significantly across CMI groups (P < 0.001). The MACE rates were 4.7% (3 cases), 7.8% (5 cases), and 30.2% (19 cases) in the low, medium, and high CMI groups, respectively, indicating a substantially elevated cardiovascular risk among patients with the highest CMI.

**Table 1:** Baseline characteristics of AF patients grouped by cardiometabolic index tertiles.

Characteristics	Tertile 1	Tertile 2	Tertile 3	P-value
CMI	0.43 (0.32-0.53)	0.85 (0.72-0.98)	1.49 (1.22-1.99)	< 0.01*
Age	74.00 (70.00-77.00)	73.50 (67.75-76.00)	72.00 (67.00-75.00)	0.32
Height(cm)	170.00 (162.00-172.00)	170.00 (161.00-172.00)	166.00 (160.00-172.00)	0.23
Weight(kg)	68.00 (62.75-72.00)	68.00 (60.00-70.00)	67.00 (62.00-70.50)	0.79
Waist(cm)	82.72 ± 5.38	83.47 ± 6.00	85.52 ± 5.06	0.013*
Hip(cm)	94.00 (91.75-95.00)	93.00 (90.75-95.00)	95.00 (92.00-96.50)	0.022*
BMI	23.90 (22.98-25.35)	23.66 (22.58-24.63)	24.31 (23.28-25.51)	0.07
FPG	5.60 (5.05-6.72)	5.70 (5.30-6.20)	6.00 (5.40-6.95)	0.09
Glycated hemoglobin	5.85 (5.40-6.50)	5.75 (5.50-6.38)	6.00 (5.60-6.65)	0.31
TC	4.59 (4.21-5.01)	4.95 (4.64-5.23)	5.13 (4.58-5.33)	< 0.01*
TG	1.02 (0.85-1.20)	1.75 (1.57-1.88)	2.31 (2.11-2.96)	< 0.01*
LDL-C	2.67 (2.33-3.09)	3.01 (2.45-3.22)	3.02 (2.52-3.44)	0.014*
HDL-C	1.20 (1.16-1.26)	1.01 (0.96-1.06)	0.82 (0.78-0.86)	< 0.01*
Hemoglobin	136.50 (131.00-150.00)	136.50 (125.00-143.25)	138.00 (129.00-151.00)	0.33
RBC	4.62 (4.24-4.93)	4.60 (4.27-4.84)	4.62 (4.47-4.90)	0.56
Hematocrit	41.95 (38.00-44.57)	41.00 (37.70-43.00)	41.00 (37.50-44.00)	0.46
Platelets	162.00 (147.00-204.50)	182.50 (152.50-213.50)	201.00 (162.50-234.50)	< 0.01*
Alanine Aminotransferase	20.00 (16.75-27.50)	22.00 (18.00-27.25)	23.00 (19.00-29.50)	0.17
Aspartate Aminotransferase	25.00 (20.00-29.50)	24.00 (20.75-31.00)	24.00 (20.50-31.50)	0.77
TotalBilirubin	17.80 (14.00-21.12)	17.20 (13.15-19.20)	16.90 (13.00-20.00)	0.27
SerumCreatinine	78.00 (68.00-89.00)	72.50 (67.00-82.25)	74.00 (63.00-88.00)	0.28
Ejection Fraction	60.00 (57.00-62.25)	61.00 (59.00-63.00)	61.00 (56.50-63.00)	0.46
Left Atrial Internal Diameter	43.00 (40.00-44.00)	42.00 (39.00-45.00)	42.00 (41.00-45.00)	0.67
Follow-up Duration (days)	711.00(394.50-904.00)	588.00 (330.00-818.75)	741.00 (482.00-944.50)	0.41
Gender (female)	39 (60.9%)	36 (56.2%)	37 (58.7%)	0.87
Radiofrequency Ablation	1 (1.6%)	2 (3.1%)	4 (6.3%)	0.31
Anticoagulant Use	46 (71.9%)	44 (68.8%)	38 (60.3%)	0.36
AF Type (Paroxysmal)	34 (53.1%)	30 (46.9%)	29 (46.0%)	0.68
CAD	14 (21.9%)	22 (34.4%)	18 (28.6%)	0.29
Diabetes	18 (28.1%)	14 (21.9%)	22 (34.9%)	0.26
Hypertension	28 (43.8%)	36 (56.2%)	34 (54.0%)	0.32
CKD	10 (15.6%)	9 (14.1%)	9 (14.3%)	0.96
Heart Failure	7 (10.9%)	7 (10.9%)	7 (11.1%)	0.99
Stroke	1 (1.6%)	2 (3.1%)	5 (7.9%)	0.18
MACE	3 (4.7%)	5 (7.8%)	19 (30.2%)	< 0.01*

* indicates statistical significance.

### Association between CMI and MACE

**Figure 2:**
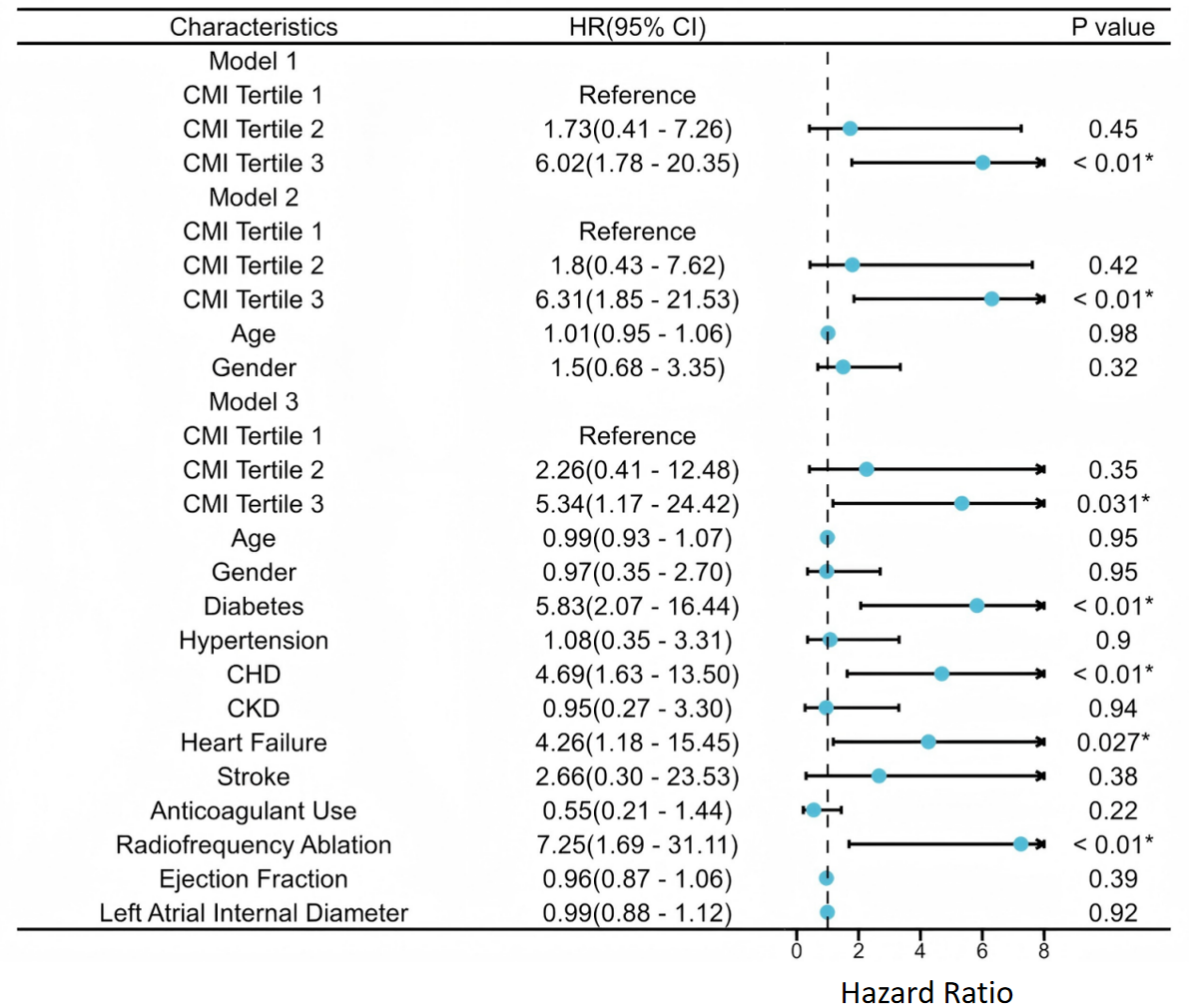
Forest plot of three Cox regression models. *The P-value show statistical significance.

**Figure 3:**
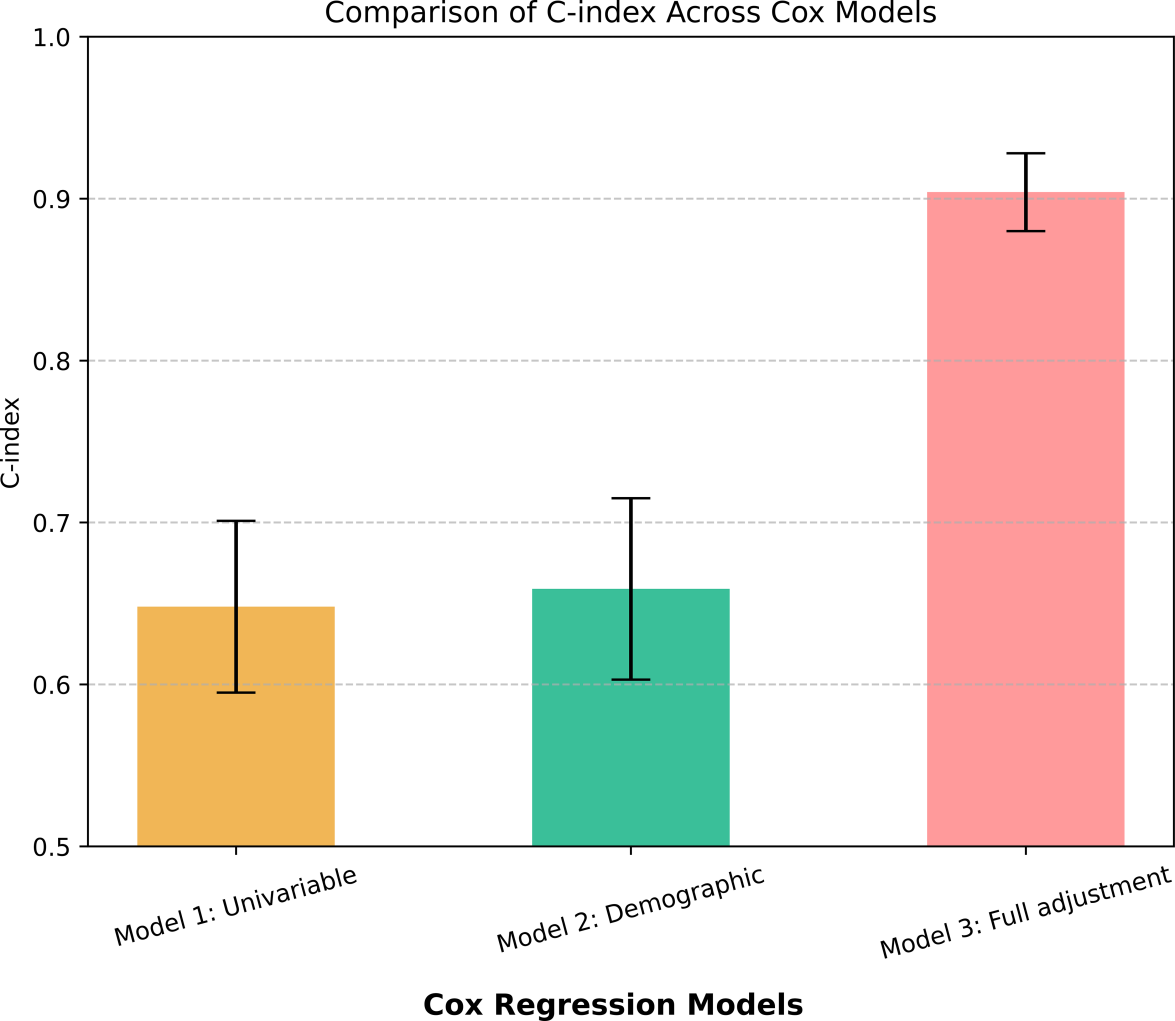
Comparison of C-index values for the three models.

To examine the relationship between CMI and the risk of MACE, three Cox proportional hazards regression models were constructed sequentially. The results are shown in **Table 2** and **Figure 2**. In the unadjusted model, patients in the high CMI tertile had a significantly elevated risk of MACE compared to those in the low CMI group (HR = 6.02, 95% CI: 1.78–20.35, P = 0.004), whereas the medium CMI group showed no significant difference (P = 0.45). After adjustment for demographic variables (age and sex), the high CMI group continued to exhibit significantly increased MACE risk (HR = 6.31, 95% CI: 1.85–21.52, P = 0.003), while age and sex themselves were not statistically significant predictors (P > 0.05). The medium CMI group remained non-significant (P = 0.42). In the fully adjusted model, which incorporated additional covariates including comorbid conditions (hypertension, CAD, CKD, heart failure, prior stroke), the high CMI group retained a significantly elevated risk of MACE (HR = 5.56, 95% CI: 1.48–20.90, P = 0.011). The medium CMI group demonstrated a non-significant upward trend (P = 0.64), reinforcing high CMI as an independent risk factor for adverse cardiovascular outcomes. Other variables identified as significant predictors of MACE in the final model included diabetes (HR = 4.60, 95% CI: 1.74–12.13, P < 0.01), coronary artery disease (HR = 4.16, 95% CI: 1.59–10.89, P < 0.01), heart failure (HR = 5.79, 95% CI: 2.06–16.30, P < 0.01). Regarding model performance, the C-index—a measure of discrimination—was 0.648 ± 0.053 for Model 1, 0.659 ± 0.056 for Model 2, and increased substantially to 0.882 ± 0.029 for the fully adjusted Model 3 (**Figure 3**), indicating excellent accuracy in differentiating high- and low-risk patients.

**Table 2:** Three Cox regression models for the association between CMI and MACE.

Characteristics	HR	95% CI	P-value
Model 1			
CMI Tertile 1	Reference		
CMI Tertile 2	1.73	0.41 - 7.26	0.45
CMI Tertile 3	6.02	1.78 - 20.35	< 0.01*
Model 2			
CMI Tertile 1	Reference		
CMI Tertile 2	1.80	0.43 - 7.62	0.42
CMI Tertile 3	6.31	1.85 - 21.53	< 0.01*
Age	1.01	0.95 - 1.06	0.98
Gender	1.50	0.68 - 3.35	0.32
Model 3			
CMI Tertile 1	Reference		
CMI Tertile 2	1.43	0.32 - 6.48	0.64
CMI Tertile 3	5.56	1.48 - 20.90	0.011*
Age	0.99	0.93 - 1.06	0.87
Gender	0.97	0.39 - 2.42	0.94
Diabetes	4.60	1.74 - 12.13	< 0.01*
Hypertension	1.28	0.44 - 3.40	0.71
CAD	4.16	1.59 - 10.89	< 0.01*
CKD	0.92	0.29 - 2.87	0.89
Heart Failure	5.79	2.06 - 16.30	< 0.01*
Stroke	1.01	0.14 - 7.39	0.99

* indicates statistical significance.

### Kaplan–Meier analysis and restricted cubic spline

**Figure 4:**
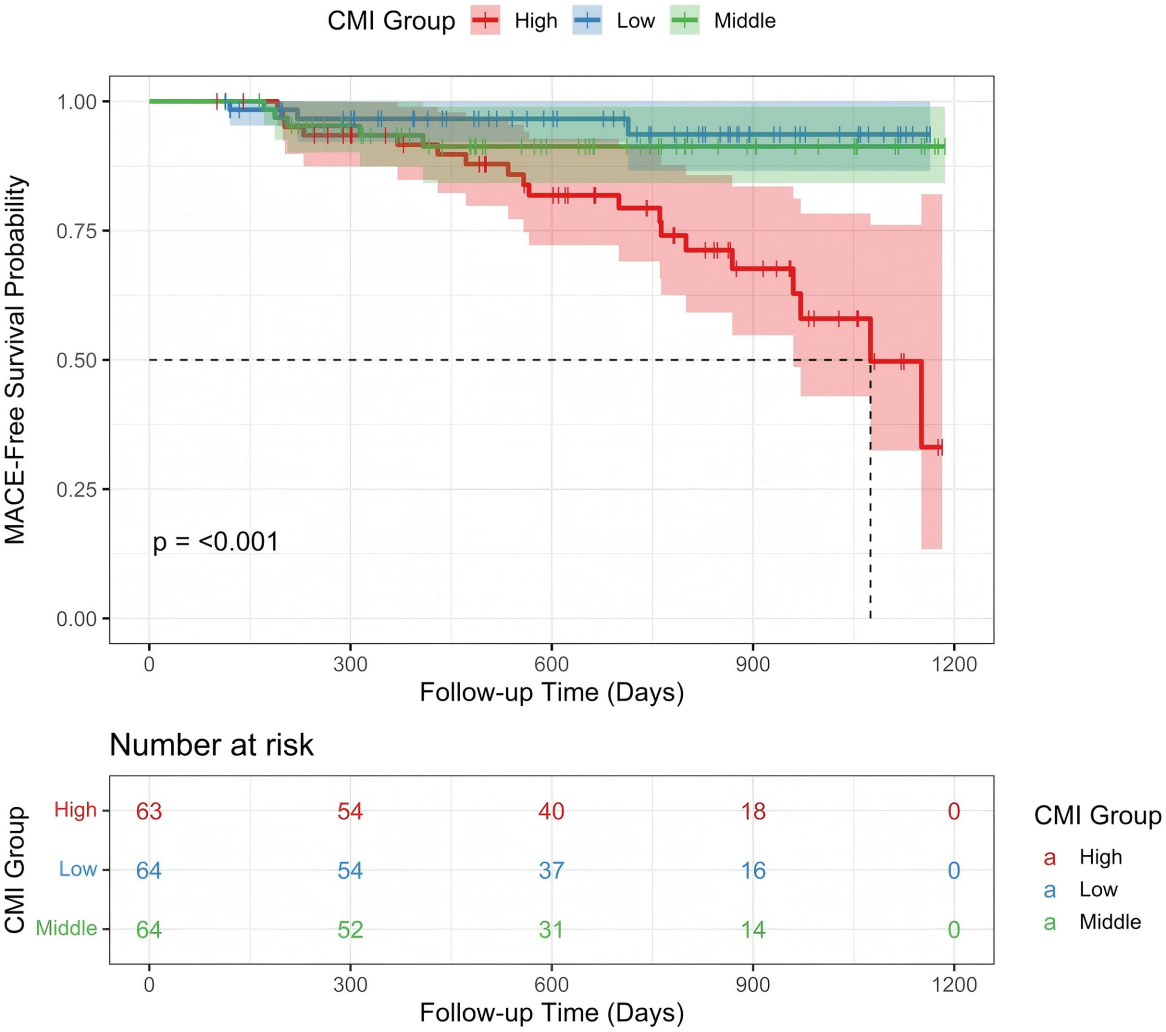
Kaplan–Meier analysis.

**Figure 5:**
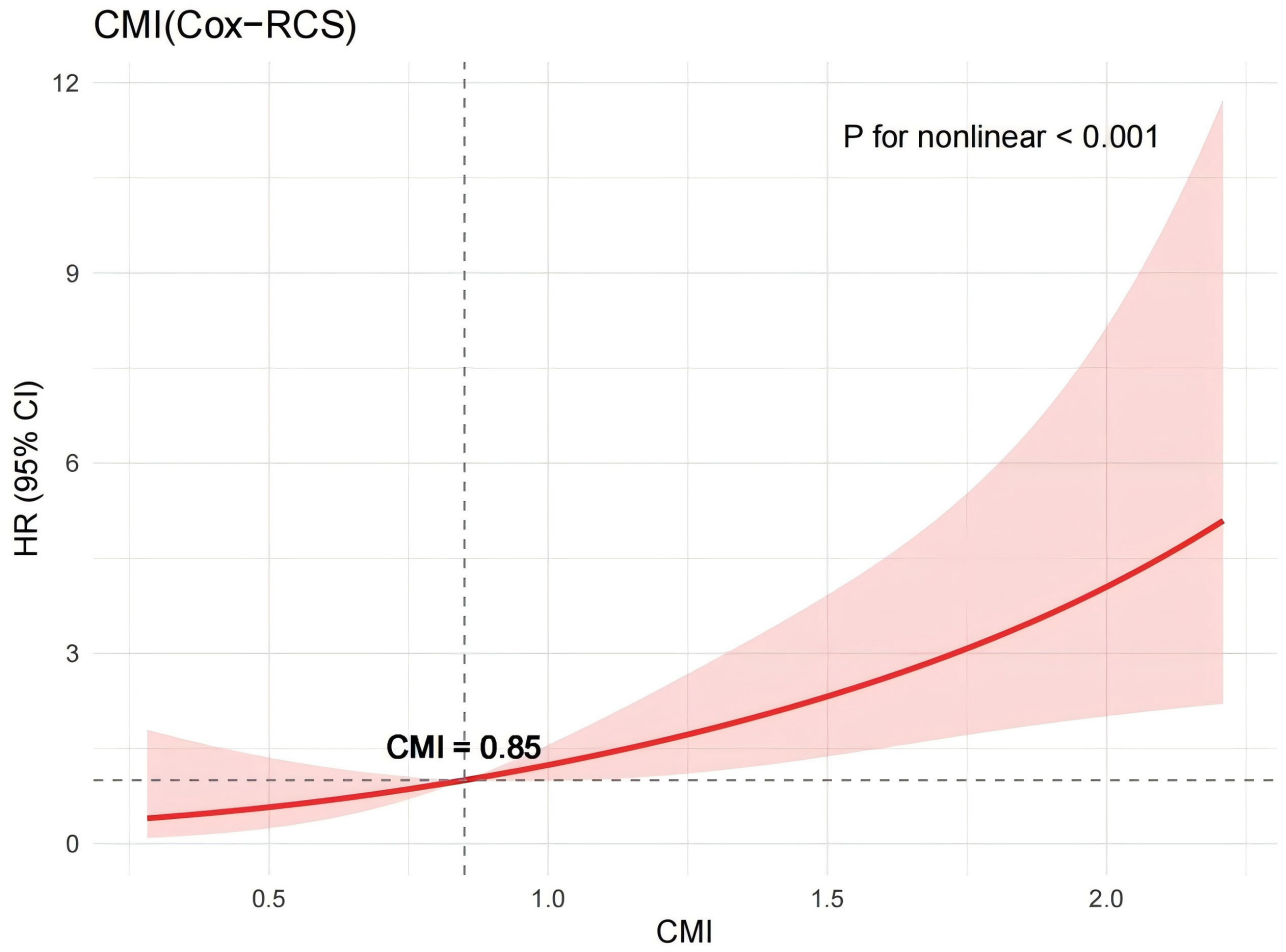
Restricted Cubic Spline.

To further explore the association between CMI levels and MACE risk, Kaplan–Meier survival curves were plotted, and differences in event rates among the three groups were assessed using the Log-rank test (**Figure 4**). Over the follow-up period, the cumulative incidence of MACE increased progressively across the CMI tertiles. The high CMI group exhibited the highest cumulative incidence, followed by the medium and low groups. The Log-rank test confirmed that the differences among the survival curves were statistically significant (P < 0.001). Throughout the observation period, the low CMI group consistently showed the lowest risk of events, the medium group displayed an intermediate risk, and the high CMI group experienced the greatest concentration of events. These findings suggest a positive correlation between higher CMI levels and increased MACE risk. To investigate the potential nonlinear relationship between CMI and MACE, a RCS model was constructed (**Figure 5**). The analysis revealed a nonlinear positive association between CMI and MACE risk (P for nonlinearity < 0.001). An inflection point was identified at approximately CMI = 0.85. Below this threshold, the risk increased gradually; beyond 0.85, the risk rose sharply and continued to escalate with increasing CMI levels. This nonlinear dose-response relationship highlights the potential threshold effect of CMI in predicting cardiovascular risk.

### XGBoost model and SHAP interpretation in machine learning

**Figure 6:**
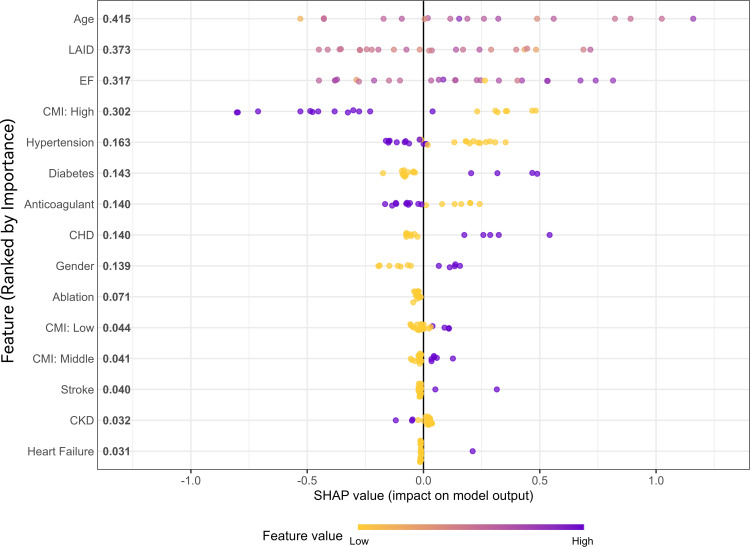
Feature importance based on SHAP values for the predictive model.

To further enhance the predictive accuracy for MACE outcomes and investigate the relative importance of input features, an XGBoost model was developed. The entire dataset was randomly divided into a training set and a testing set at a ratio of 8:2. Model hyperparameters were optimized using ten-fold cross-validation within the training set. In terms of predictive performance, the XGBoost model achieved a C-index of 0.737 on the testing set. While this performance exceeded that of the unadjusted and demographic-only Cox regression models, it remained lower than that of the fully adjusted Cox model. To improve model interpretability, SHAP were applied to quantify the marginal contribution of each variable to the model’s output (**Figure 6**). Based on mean SHAP values, the top four predictors were age (mean SHAP: 0.415), LAID (0.373), LVEF (0.317), and high CMI category (0.302), indicating that both structural cardiac parameters and metabolic status significantly influenced model predictions. Other key contributors included hypertension, diabetes, anticoagulation therapy, and coronary artery disease.

### Subgroup analysis

**Figure 7:**
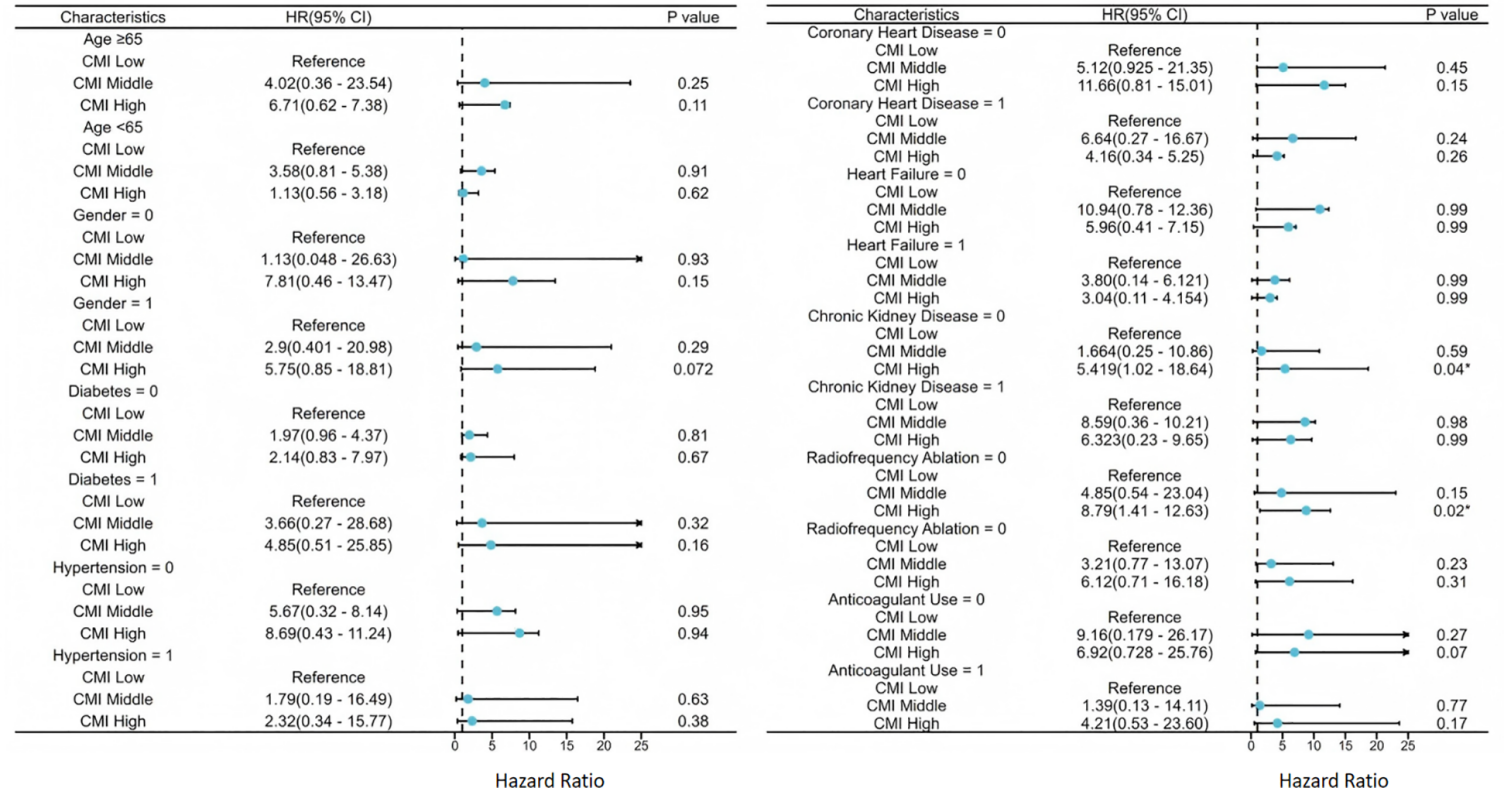
Association between CMI and impaired MACE by subgroup. *The P-value show statistical significance.

To further assess the prognostic value of CMI for MACE across different clinical populations, multivariable Cox proportional hazards subgroup analyses were conducted based on key variables, including age, sex, hypertension, CAD, CKD, heart failure, anticoagulation therapy status, and receipt of catheter ablation (**Figure 7**). The analysis identified two subgroups in which elevated CMI was significantly associated with increased MACE risk. Among patients without CKD, those in the high CMI group had a significantly higher risk of MACE (HR = 5.42, 95% CI: 1.03–28.65, P = 0.047). Similarly, among patients who had not undergone radiofrequency ablation, high CMI was also significantly associated with MACE risk (HR = 10.8, 95% CI: 1.41–82.64, P = 0.022). No statistically significant associations between CMI and MACE were observed in the remaining subgroups (all P > 0.05).

## Discussion

This study is the first, to our knowledge, to systematically assess the relationship between CMI and MACE in a real-world, community-based cohort of patients with AF. The results demonstrated that elevated CMI levels were significantly associated with a higher risk of MACE, and this association remained robust after comprehensive multivariable adjustment. Both Kaplan–Meier analysis and RCS modeling revealed a nonlinear positive relationship, with a sharp increase in cardiovascular risk observed once CMI exceeded approximately 0.85. Additionally, subgroup analyses showed that the predictive value of CMI was particularly evident in patients without CKD and those who had not undergone catheter ablation.

While previous studies have established links between central obesity, metabolic syndrome, and AF incidence—such as findings from the Rotterdam cohort and research in patients with type 1 diabetes mellitus (T1DM)—most of these investigations have focused on individual metabolic components like BMI, glycemia, or dyslipidemia, or have relied on loosely defined metabolic phenotypes (7–10, 16). In contrast, CMI, which integrates waist-to-height ratio and triglyceride-to-HDL cholesterol ratio, represents a more comprehensive metabolic marker. Its role in AF-related risk prediction, however, has not been well explored. Although CMI has shown predictive utility in patients with coronary artery disease and diabetes, its independent contribution and added value in AF populations have remained unclear (13, 17–19). Our findings provide new evidence that incorporating CMI into MACE risk prediction models significantly enhances model performance. Specifically, the C-index increased from 0.659 in the demographic-only model to 0.904 in the fully adjusted model—a substantial improvement that underscores CMI’s potential as a powerful tool for identifying high-risk AF patients. This enhancement is particularly relevant in primary care or resource-constrained settings, where efficient pre-screening tools are essential. Moreover, by integrating nonlinear spline modeling and machine learning techniques (XGBoost with SHAP interpretation), we demonstrated the robustness and interpretability of CMI in complex clinical scenarios. This multimethod approach addresses limitations of traditional regression models, which often fail to capture intricate variable interactions and nonlinear effects. Our results suggest that the combined use of conventional and machine learning models can offer more nuanced and reliable risk stratification strategies, potentially informing future research and clinical practice.

We identified a significant nonlinear relationship between CMI and the risk of MACE, suggesting that beyond a critical threshold, metabolic disturbances may trigger a decompensated response in the cardiovascular system. This nonlinearity likely arises from the synergistic interplay of multiple metabolic pathways. First, central obesity, the core component of CMI, promotes the accumulation of visceral and pericardial fat (20–22). These adipose tissues act as active paracrine organs, secreting pro-inflammatory cytokines (e.g., IL-6, TNF-α) and reactive oxygen species, which contribute to chronic low-grade inflammation and oxidative stress (20–23). These processes jointly drive atrial remodeling—both structural and electrical. Prior research has shown that each 1 SD increase in pericardial fat thickness is associated with a 2.6-fold increased risk of atrial fibrillation (23, 24), likely mediated by inflammation spread, autonomic nervous system activation, and myocardial fibrosis. Second, an elevated TG/HDL-C ratio reflects significant lipoprotein metabolic dysfunction, favoring the generation of small, dense LDL particles that promote atherogenesis (25, 26). Concurrently, impaired HDL function compromises endothelial protection and enhances prothrombotic activity, significantly increasing the risk of thrombus formation—a critical concern in AF patients who are already predisposed to ischemic stroke and related complications (27, 28). Third, our data revealed that when CMI exceeds approximately 0.85, MACE risk rises steeply. This threshold effect may indicate that the cumulative metabolic burden surpasses the heart’s compensatory capacity, resulting in overt atrial structural remodeling (e.g., atrial dilation, fibroblast activation) and electrical abnormalities (e.g., P-wave prolongation, heightened ectopic activity). These structural-electrical changes likely reflect a transition from adaptive to maladaptive myocardial responses (29, 30). Supporting this interpretation, prior studies have demonstrated that weight reduction can reverse atrial remodeling and reduce AF burden, underscoring the potential reversibility of these changes (31). Collectively, these findings suggest that CMI is more than a composite marker of central obesity and dyslipidemia—it may serve as a key “stress axis” integrating metabolic, inflammatory, electrophysiologic, and thrombotic pathways. Moreover, given the pathophysiological role of central obesity and lipid dysregulation in AF progression and MACE development, our findings underscore the potential value of lifestyle-based interventions in mitigating cardiometabolic risk. For instance, adherence to the Mediterranean diet—rich in anti-inflammatory and antioxidant components—has been shown to improve metabolic profiles and reduce cardiovascular events among AF patients (32). These dietary patterns may help attenuate visceral adiposity, improve HDL functionality, and suppress systemic inflammation, thereby potentially modulating the CMI and its downstream consequences. Integrating such non-pharmacological strategies into AF management may offer a practical and effective approach to lowering MACE risk, particularly in primary prevention settings.

The results of this study suggest that the CMI, as a simple and readily accessible composite metabolic indicator, may serve as a practical tool for risk stratification in patients with AF. By capturing both central obesity burden and lipid metabolic dysfunction, CMI provides additional prognostic information beyond traditional scoring systems such as CHA_2_DS_2_-VASc. Its ease of calculation and broad availability make it particularly well-suited for use in primary care settings or resource-limited community health platforms, where it could aid in the early identification of high-risk individuals, guide anticoagulation decision-making, and inform referral strategies. Furthermore, exploratory modeling using machine learning techniques—specifically XGBoost combined with SHAP interpretation—demonstrated that CMI ranked among the most important predictors in complex, nonlinear frameworks. These findings underscore CMI’s modeling stability and interpretability, supporting its future integration into intelligent risk prediction systems designed to enhance precision clinical decision-making.

Nonetheless, this study has several limitations. First, it was a single-center retrospective analysis with a modest sample size, which may introduce selection bias and data incompleteness. Second, the study did not include measurements of key mechanistic biomarkers related to inflammation, oxidative stress, or insulin resistance, limiting insight into the biological pathways linking CMI to cardiovascular outcomes. Third, the relatively short median follow-up period may not fully capture the long-term dynamic relationship between CMI and MACE. Therefore, future research should aim to validate these findings in larger, multicenter, prospective cohorts with extended follow-up durations to confirm their robustness and generalizability in diverse clinical settings.

Future research may be expanded in the following three areas: First, by integrating mechanistic biomarkers such as inflammatory cytokines, adiponectin levels, and insulin sensitivity indices, researchers can systematically elucidate the metabolic–inflammatory–electrophysiological pathways underlying CMI and clarify its biological foundation. Second, large-scale datasets can be used to construct CMI-based risk scoring systems and compare them with existing tools such as the CHA_2_DS_2_-VASc score to assess reclassification capability and incremental predictive value. Third, further exploration is needed to determine the clinical utility of CMI in guiding AF management—particularly in selecting anticoagulation strategies, timing catheter ablation, and implementing lifestyle interventions—thus facilitating the development of a closed-loop risk management model that connects risk prediction with targeted intervention.

## Conclusions

This study demonstrates that elevated CMI levels are significantly associated with increased risk of MACE in patients with atrial fibrillation, exhibiting a nonlinear trend. CMI remained an independent predictor even after adjustment for multiple clinical risk factors. As a simple and readily available composite metabolic indicator, CMI may aid community healthcare providers in stratifying cardiovascular risk and guiding early intervention planning in the management of atrial fibrillation.

## Data Availability

The raw data supporting the conclusions of this article will be made available by the authors, without undue reservation.
